# Accuracy Improvement of a Compact ^85^Rb Atom Gravimeter by Suppressing Laser Crosstalk and Light Shift

**DOI:** 10.3390/s23136115

**Published:** 2023-07-03

**Authors:** Guiguo Ge, Xi Chen, Jinting Li, Danfang Zhang, Meng He, Wenzhang Wang, Yang Zhou, Jiaqi Zhong, Biao Tang, Jie Fang, Jin Wang, Mingsheng Zhan

**Affiliations:** 1 Innovation Academy for Precision Measurement Science and Technology, Chinese Academy of Sciences, Wuhan 430071, China; 2 University of Chinese Academy of Sciences, Beijing 100049, China; 3 Hefei National Laboratory, Hefei 230094, China; 4 Wuhan Institute of Quantum Technology, Wuhan 430206, China

**Keywords:** atom gravimeter, compact, accuracy, stability

## Abstract

We design and implement a compact ^85^Rb atom gravimeter (AG). The diameter of the sensor head is 35 cm and the height is 65 cm; the optical and electronic systems are installed in four standard 3U cabinets. The measurement accuracy of this AG is improved by suppress laser crosstalk and light shift. In addition, the angle of the Raman laser reflector is adjusted and locked, and the attitude of the sensing head is automatically adjusted, and the vibration noise is also compensated. The comparison measurement results between this AG and the superconducting gravimeter indicate that its long-term stability is 0.65 μGal @50000 s.

## 1. Introduction

The atom gravimeter (AG) is a kind of absolute gravity measurement instrument. It uses laser-cooled cold atoms as the measurement mass, and has the advantages of no measurement drift and a high-measurement rate. It has important usage in the field of geophysics [1], resource exploration [2], seismology [3], and navigation [4]. In 1992, Kasevich et al. realized the gravity measurement by an atom interferometer (AI) [5]. In 1999, Peters et al. achieved an AI-based gravity measurement with a precision of 10^−9^g and compared the measurement value with that of an FG-5 falling corner-cube gravimeter [6]. In 2013, Hu et al. developed an ultrahigh-sensitivity AG, and achieved a sensitivity of 4.2 μGal/$\sqrt{\mathrm{Hz}}$ [7]. In 2016, Freier et al. realized an AG with a long-term stability of 0.5 nm/s^2^ [8]. Several AGs participated the International Comparison of Absolute Gravimeters (ICAG) [9,10,11]. New measurement methods for the AG have also been proposed, including the Bloch oscillation [12], the double diffraction Raman transition [13], the point source acceleration measurement [14], the high data-rate atom interferometry [15], and clarification of relevant systematic error terms [16,17,18]. Most practical application scenarios require portable AGs. Menoret et al. realized a compact AG [19] with a sensor head of 70 cm in height and long-term stability of 1 μGal. Deng et al. developed two compact atom gravimeters named as RAI g and MAIN [20] with a sensitivities of 15 μGal/$\sqrt{\mathrm{Hz}}$ and 1.9 m Gal/$\sqrt{\mathrm{Hz}}$, respectively. Other groups also made efforts towards the compact and practical design of AGs [21,22,23,24].

However, the portability of an AG is contradictory to measurement accuracy. How to improve measurement accuracy is an important issue and extremely challenging task facing the application of miniaturized AGs. For instance, the systematic error caused by tilt is a formidable issue for the practical application of gravimeters; the systematic errors of light shift imbalance [23] could not be eliminated just by interleaving the direction of *k*_eff_; and the optical system using the electro-optic modulation(EOM) scheme exhibits the crosstalk effect, which can affect the measurement accuracy.

We design and develop a compact AG using ^85^Rb atoms. It consists of a sensor head, optical unit, and electronic unit. The diameter of the sensor head is 35 cm and the height is 65 cm. The optical unit adopts a single-seed laser design which is quite different from Refs. [19,20]. The optical and electronic systems are installed in four standard 3U cabinets. To improve the measurement accuracy of this AG, we have taken measures to suppress laser crosstalk and light shift. By optimizing the position and interference timing of the Raman laser reflector, we suppressed the crosstalk of the higher-order harmonics of the lasers. By optimizing and locking the sideband-ratio of the Raman laser pulse, we eliminated ac Stark shift. To guarantee the consistency of the gravity and Raman laser’s direction, the angle of the Raman laser reflector is adjusted and locked through a piezoelectric ceramic (PZT) driving mirror frame, and the attitude of the sensing head is automatically adjusted using an electronic control bracket. In addition, the vibration noise is compensated during the measurement. We conducted gravity comparison measurements between this AG and a superconducting gravimeter, and the results shows that the long-term stability of the AG is 0.65 μGal@50000 s.

## 2. Process Design for Measuring Gravity Using ^85^Rb Atoms

**Figure 1:**
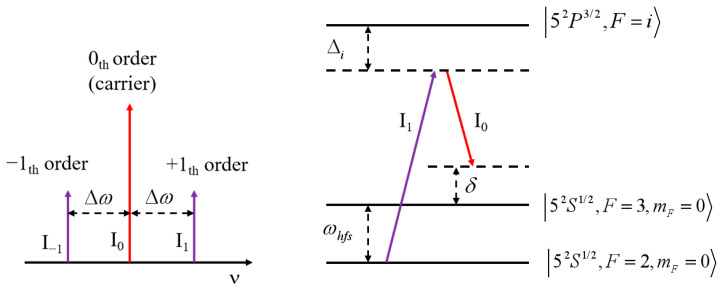
Raman transition of the ^85^Rb atom by using lasers created by the sidebands of fiber-EOM.

The gravimeter utilizes the free-falling cold ^85^Rb atoms, and utilizes the stimulated Raman transition to achieve the interference process, as can be found in Ref. [25]. The lasers for cooling, repump, optical pumping, Raman operation and detection are created by the sidebands of a fiber-EOM. An illustration of the sidebands of the Raman laser and Raman transition are shown in Figure 1. The carrier frequency of the seed laser can be tuned for more than 1 GHz by using the sideband frequency locking method, and the detail information can be found in Ref. [26]. In the laser cooling stage, the laser frequency is red-detuned (−10 MHz) to the |5^2^S_1/2_, *F* = 3〉 → |5^2^P_3/2_, *F* = 4〉 transition, while the repump laser is in resonance with the |5^2^S_1/2_, *F* = 2〉 → |5^2^P_3/2_, *F* = 3〉 transition. Then, the polarization gradient cooling (PGC) process is used by varying the frequency and intensity of the cooling laser. Most of the atoms are then populated in the |5^2^S_1/2_, *F* = 3〉 state. The atomic cloud has about 10^7^ atoms with 1D temperature of 3 μK. Before the interference process, a laser pulse, which is in resonance with the |5^2^S_1/2_, *F* = 3〉 → |5^2^P_3/2_, *F* = 3〉 transition, is applied, then the atoms are pumped to the |5^2^S_1/2_, *F* = 2〉 state. In the Raman interference process, the π/2-π-π/2 Raman pulses act with atoms in sequence. The first π/2 pulse is applied to split the cold atom cloud to the superposition state of |5^2^S_1/2_, *F* = 2, m_F_ = 0〉 state and |5^2^S_1/2_, *F* = 3, m_F_ = 0〉 state; after T = 71 ms, a π pulse is applied to refocus the two coherent states; after another T = 71 ms, the second π/2 pulse is applied to recombine the coherent states and generate interference. The single photon detuning of the Raman laser is −752 MHz to the |5^2^S_1/2_, *F* = 3〉 → |5^2^P_3/2_, *F* = 4〉 transition, and the two-photon detuning δ in Figure 1 is turned to compensate the Doppler frequency shift, which is caused by the free fall of the atoms. The time duration of the Raman π pulse is 20 μs. The interference fringe is obtained in the fluorescence detection stage. The normalized fluorescence detection is used to eliminate the noise caused by fluctuations of the atom number and variation of the light intensity. The detection laser is tuned to be in resonance with the |5^2^S_1/2_, *F* = 3〉 → |5^2^P_3/2_, *F* = 4〉 transition, and applied to the detection laser pulse for 4 ms. Then, the fluorescence emitted from the atoms in |5^2^S_1/2_, *F* = 3〉 state is recorded. The intensity of the fluorescence is labeled as *P*_1_. Then, the repump pulse is turned on for 0.1 ms to pump the atoms from |5^2^S_1/2_, *F* = 2〉 state to |5^2^S_1/2_, *F* = 3〉 state, and the detection laser pulse is turned on for another 4 ms to excite the fluorescence. The intensity of fluorescence is labeled as *P*_2_, *P* = *P*_1_/*P*_2_ represents the population in |5^2^S_1/2_, *F* = 3〉 state. The interference population *P* has the following relationship:

(1) $$P=A\left[1+C\cos\phi \right]=A\left[1+C\cos[\left(k_{eff}g-2\pi \alpha \right)\left(T+\tau \right)\left(T+2\tau /\pi \right)\right]],$$

where *ϕ* is the phase of the interference fringe, *g* is the value of gravity, *α* is the chirp rate of the Raman laser, *τ* is the duration of the π pulse, *T* is the free evolving time, *A* is the offset and *C* is the contrast of fringe. The value of *A*, *C* and *ϕ* can be obtained by the sine curve fitting of *P*, and the value of gravity can by derived from the value of *ϕ*. The wave vectors inversion method [27] is adopted to suppress the phase errors which are independent with the Raman laser’s wave vector, such as the fringe phase shift caused by the Zeeman shifts and ac Stark shifts.

## 3. Implementation of the Compact ^85^Rb Atom Gravimeter

**Figure 2:**
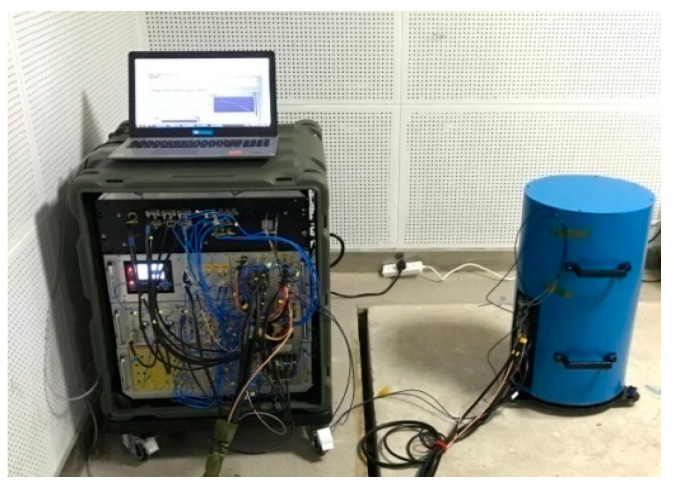
The Photo of the compact ^85^Rb atom gravimeter.

The compact ^85^Rb AG is shown in Figure 2. It consists of a sensor head, an optical system and electronic system. The sensor head has a size of *ϕ*35 cm × 65 cm, and a weight of 42 kg. The optical system and electronic system are placed in four standard 3U chassis, and then installed in a standard 12U cabinet. The sensor head and the cabinet are connected by a 5 m long cable. The power consumption of the AG is about 250 W.

### 3.1. The Sensor Head

**Figure 3:**
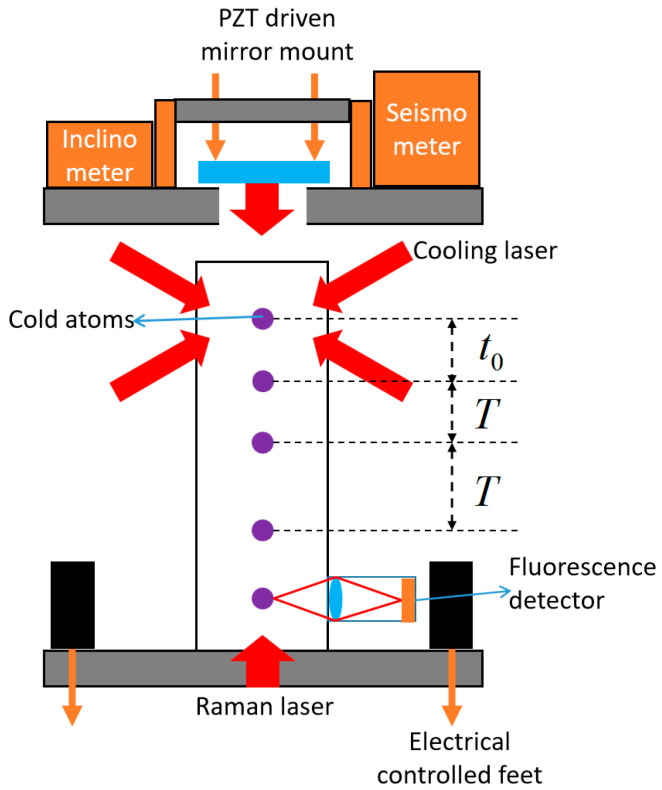
Scheme diagram of the sensor head.

The scheme diagram of the sensor head is shown in Figure 3. The core of the sensor head is a titanium vacuum chamber which employs the indium sealing technology.

The vacuum of the chamber is maintained by a 2 L ion pump and a getter. The vacuum has a level of 10^−8^ Pa. The rubidium atoms are provided by a dispenser installed in the upper part of the chamber. Two single-mode polarization-maintaining (SMPM) fibers are used to connect the sensor head and the optical system. A laser from one fiber passes through a beam expander and is divided into two paths. They form two pairs of horizontal laser beams which act as the horizontal cooling lasers. A laser from another fiber passes through a beam expander, a liquid crystal variable retarder (LCVR), a series of reflecting mirrors, and propagates through the vacuum chamber in a vertical upward direction. The laser is then reflected by a reflecting mirror mounted on the top of the sensor head, forming a vertical propagated laser pair. This laser pair acts as the vertical cooling laser, the Raman laser, and the fluorescence detection laser by adjusting the frequency of the laser and the phase of the LCVR.

The disagreement among the directions of *g* and Raman beams results in an alignment error. In order to adjust the Raman laser angle accurately, we design and adopt two angle adjustment units. The first is a mirror mount that can be adjusted manually or by a PZT driver to change the angle of the Raman laser mirror. The second is an electrically controlled bracket with one fixed foot and two controlled feet based on stepper motors. It works together with a high-precision digital inclinometer inside the sensor head to adjust its attitude.

The vibration will result in phase noise in the interference fringe, thus reducing the precision of the gravity measurement. We installed a seismometer on the top of the sensor head to measure vibration signals in real time, and the signals are used to compensate the vibration noise in gravity measurement.

### 3.2. The Optical System

The design of the optical system is based on our previous work [26], and several improvements have been taken. The output of a seed laser is divided into two paths, one of which passes through a fiber-EOM (labeled as FEOM1) and is injected to a saturated absorption module. The +1-order sideband of the output laser of the FEOM1 is lock to the ^87^Rb |5^2^S_1/2_, *F* = 2〉 to |5^2^P_3/2_, *F* = co (2,3)〉 transition. By adjusting the driving frequency of FEOM1, the carrier frequency of the seed laser can be adjusted. The other path of the seed laser passes through another fiber-EOM (labeled as FEOM2) to create laser sidebands. By adjusting the driving frequencies of FEOM1 and FEOM2, the output sidebands of the FEOM2 can realize various operations for ^85^Rb atoms, such as laser cooling, repumping, Raman transition, and fluorescence detection. The output laser of FEOM2 is amplified by a tapered amplifier (TA). A small fraction of the output laser is coupled into an Fabry–Perot (FP) cavity to measure the power of each sideband, while most of the laser is coupled to an acousto-optic modulator (AOM) to control the laser amplitude. The output laser of AOM is divided into two paths, and then coupled into two SMPM fibers.

To improve the stability and maintainability of the optical system, we divide the optical system into five independent optical modules, each module is connected by SMPM fibers. We placed these modules into a chassis, and used heating films and a temperature control circuit to control the temperature of the chassis above the ambient temperature; the chassis is surrounded with thermal insulation material to reduce the power consumption of the circuit and increase the temperature control precision. The temperature fluctuation of the optical system is controlled to be less than 0.03 °C, and the corresponding variation of the output laser intensity is less than 1%.

### 3.3. The Electronic System

The electronic system consists of the power supply unit, the control unit, and the functional circuits. The power supply unit adopts a combination of linear power supply and switch power supply. The linear power supply is used to drive the seed laser and TA, as well as the fluorescence amplification circuits. The switch power supply is used to drive the MOT-coil, the dispenser, the temperature control circuits and the microwave circuits. A control module (NI-CRIO 6038) is used to achieve the AO control, DIO control, and AI acquisition function. The functional circuits include the laser current control circuit, the laser frequency locking circuit, the temperature control circuit, the current control circuit, the photoelectric amplification circuit, and the microwave generation circuits. In order to improve the stability of microwave frequency, a rubidium atomic clock is used as the time standard. All modules are integrated into three standard 3U chassis and then installed in a 12U cabinet. All serial port signals and USB signals are connected to a USB-HUB and then to a computer with a single USB cable.

## 4. Measures to Improve the Accuracy of the Compact ^85^Rb AG

In order to improve the measurement accuracy of this compact AG, we have taken four measures, including automatic tilt adjustment of the attitude of the sensing head, suppressing the light shift, suppressing the crosstalk of additional laser lines, and compensation of vibration noise.

### 4.1. Automatic Tilt Adjustment

**Figure 4:**
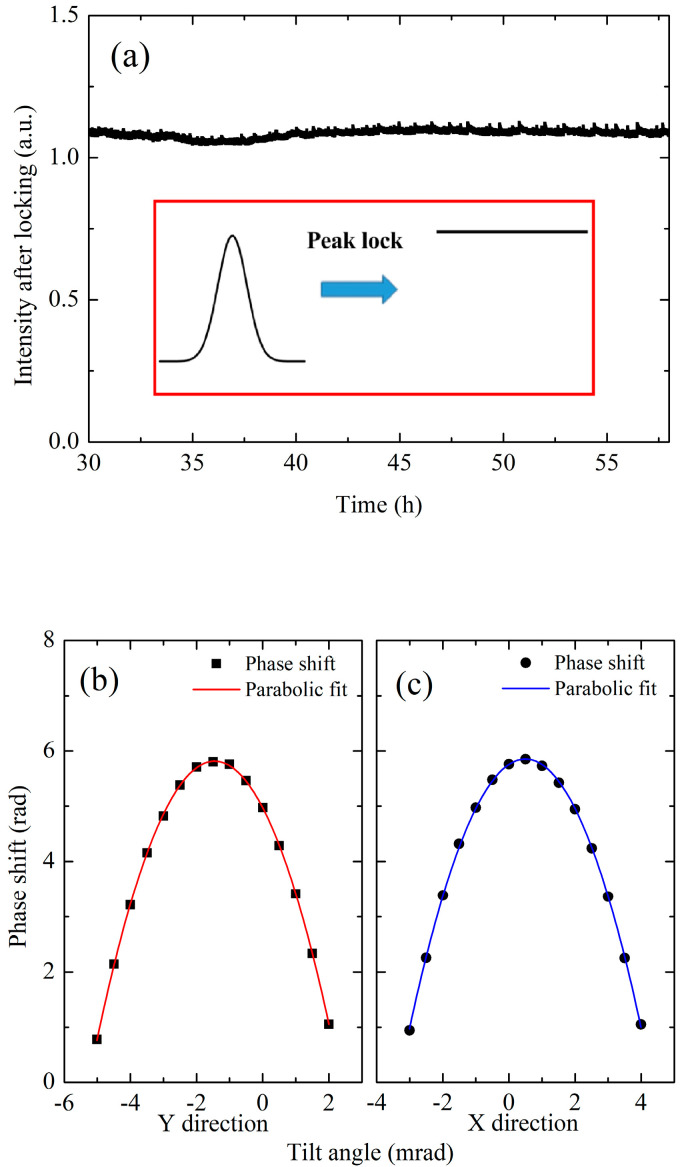
Experimental results of tilt adjustment. (**a**) Peak lock of the intensity of the reflect Raman laser coupled to the SMPM fiber; the insert picture represents the process of peak lock. (**b**,**c**) measured the phase shift of the AG by scanning the attitude angle of the sensor head. The red solid line represents Y direction and the blue solid line represents X direction.

Firstly, we used a mirror mount that can be adjusted both manually and by a PZT driver, to adjust the angle of the reflecting mirror of the Raman laser. The reflect angles of the Raman laser are labeled as *θ*_x_ and *θ*_y_. By adjusting these two angles, we can reflect the Raman laser to its incident direction, and coupled it back to the incident SMPM fiber; the power of the reflect laser is then detected by a photodetector (PD) installed in the optical system, labeled as *I*_R_. During the gravity measurement process, we modulated the reflect angles. By demodulating the power of *I*_R_, and feeding back the error signal to the reflect angles, we realize a peak lock of the *I*_R_ to its maximum value, thus ensuring the overlapping of Raman laser pairs, as shown in Figure 4a. The real-time controlled angle stability is established by the demodulation signal of *I*_R_ after locking the associated with the linewidth of *I*_R_ with the adjusted angles before locking. The overlapping of Raman laser pairs after locking is established to be better than 3 μrad. Secondly, we design an electrical controlled bracket to adjust the attitude of the sensor head, the electrical controlled bracket has one fix foot and two electrical controlled feet, which are based on stepper motors. The bracket works are associated with a high-precision digital inclinometer installed inside the sensor head. We adopted the traditional PID locking method, which feeds back the angle errors of the inclinometer to the stepper motors to achieve closed-loop locking. To avoid the vibration noise of the motors after locking, we set a threshold for the angle control. When the angle errors of the inclinometer are within the threshold, we stopped the feedback of the motors. The threshold can be adjusted from 0.05 mrad to 0.01 mrad, and the angle adjustment accuracy is in the same order. By scanning the attitude angle of the sensor head, we adjusted the angle between the direction of the Raman laser and the gravity. When the two directions are consistent, the measured gravity value is maximum as shown in Figure 4b,c. By using these two methods, the angle between the Raman laser and the gravity is adjusted to be less than 20 μrad; the corresponding uncertainty of gravity measurement is less than 0.2 μGal.

### 4.2. Suppressing the Light Shift

**Figure 5:**
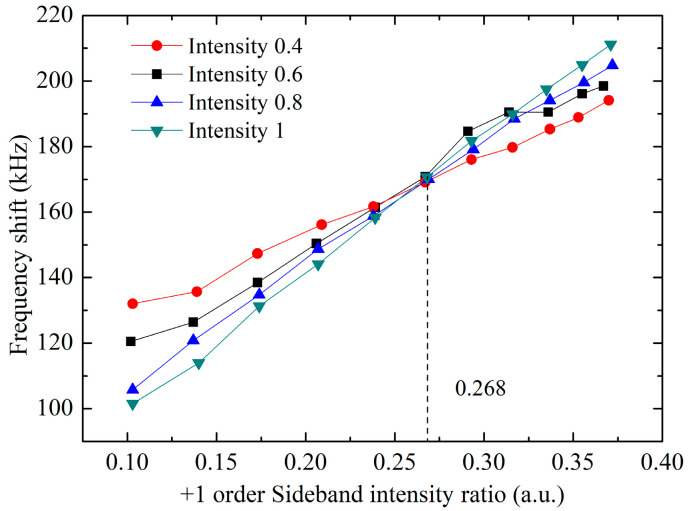
Relationship between the measured frequency shift of the Raman transition and the sideband ratio of the Raman laser. Different lines represent different Raman laser intensities.

The Raman laser contains complex sideband components due to the phase modulation. The sidebands of the Raman laser induce the ac Stark shift, which will result in the phase shift of the interference fringes [25]. Although we adopted the wave vectors inversion method to suppress the ac Stark shift, the residual phase shift still exists if the Raman laser ratios for the *k*_eff_+ and *k*_eff_− Raman transition are different. So, we managed to eliminate the ac Stark shift of the Raman laser for the multi-sidebands case. We calculated the ac Stark shift $\Omega_{2}^{\mathrm{AC}}$ for the |5^2^S^1/2^, *F* = 2, *m*_F_ = 0〉 state and $\Omega_{3}^{\mathrm{AC}}$ for the |5^2^S_1/2_, *F* = 3, *m*_F_ = 0〉 state; the differential ac Stark shift $\delta^{\mathrm{AC}}$ of these two states is:

(2) $$\delta^{\mathrm{AC}}=\Omega_{2}^{\mathrm{AC}}-\Omega_{3}^{\mathrm{AC}}=\sum_{i,j}\frac{{\left|\Omega_{i,2,j}\right|}^{2}}{4\left(\Delta_{i}-\omega_{\mathrm{hfs}}+j\Delta \omega \right)}-\sum_{i,j}\frac{{\left|\Omega_{i,3,j}\right|}^{2}}{4\left(\Delta_{i}+j\Delta \omega \right)},$$

where *i* represents the available excited states for the Raman transition, *j* = −1, 0, 1 represents the −1, 0, 1 order sidebands of the Raman laser (we ignored sidebands larger than 1 or less than −1), $\Omega_{i,2,j}$ ($\Omega_{i,3,j}$) is the Rabi frequency of the |5^2^S_1/2_, *F* = 2, *m*_F_ = 0〉 state (|5^2^S_1/2_, *F* = 3, *m*_F_ = 0〉 state) to the *i*th available 5^2^P_3/2_ state induced by the *j*th sideband, $\Delta_{i}$ is the single photon detuning of the |5^2^S_1/2_, *F* = 3, *m*_F_ = 0〉 state to the *i*th available 5^2^P_3/2_ state by the 0 order sideband, $\omega_{\mathrm{hfs}}$ is the hyperfine frequency between the |5^2^S_1/2_, *F* = 2, *m*_F_ = 0〉 and the |5^2^S_1/2_, *F* = 3, *m*_F_ = 0〉 states, and Δω is the frequency difference of the Raman laser sidebands. We labeled the laser intensity of the *i*th sidebands of the Raman laser as $I_{i}$, and the total intensity of the Raman laser as *I*. For a given single photon detuning, we calculated the sidebands ratio as $\beta =I_{-1}/I=I_{+1}/I$ that eliminates the differential ac Stark shift $\delta^{\mathrm{AC}}$. When $\Delta_{1}=-752\;\mathrm{MHz}\;$ $(\Delta_{1}$ is the single photon detuning of the |5^2^S_1/2_, *F* = 3, *m*_F_ = 0〉 to |5^2^P_3/2_, *F* = 4, *m*_F_ = 0〉 transition by the 0-order sideband, the calculated ratio β is 0.268. We carried out the experiment to measure this optimized sideband ratio. We set $\Delta_{1}$ to be −752 MHz, and scanned the two-photon detuning δ to obtain the Raman transition spectrum. We changed the sideband ratio to obtain the relationship between the central position of the spectrum and the sideband ratio. As shown in Figure 5, the intersection point of these curves represents the optimized sideband ratio for $\delta^{\mathrm{AC}}=0$; the measured sideband ratio is 0.268, which is consistent with the theoretical calculation result. At this sideband ratio, the proportion of +2 and −2 order sidebands were calculated, according to the sideband modulation intensity being 0.02, and its influence can be ignored.

**Figure 6:**
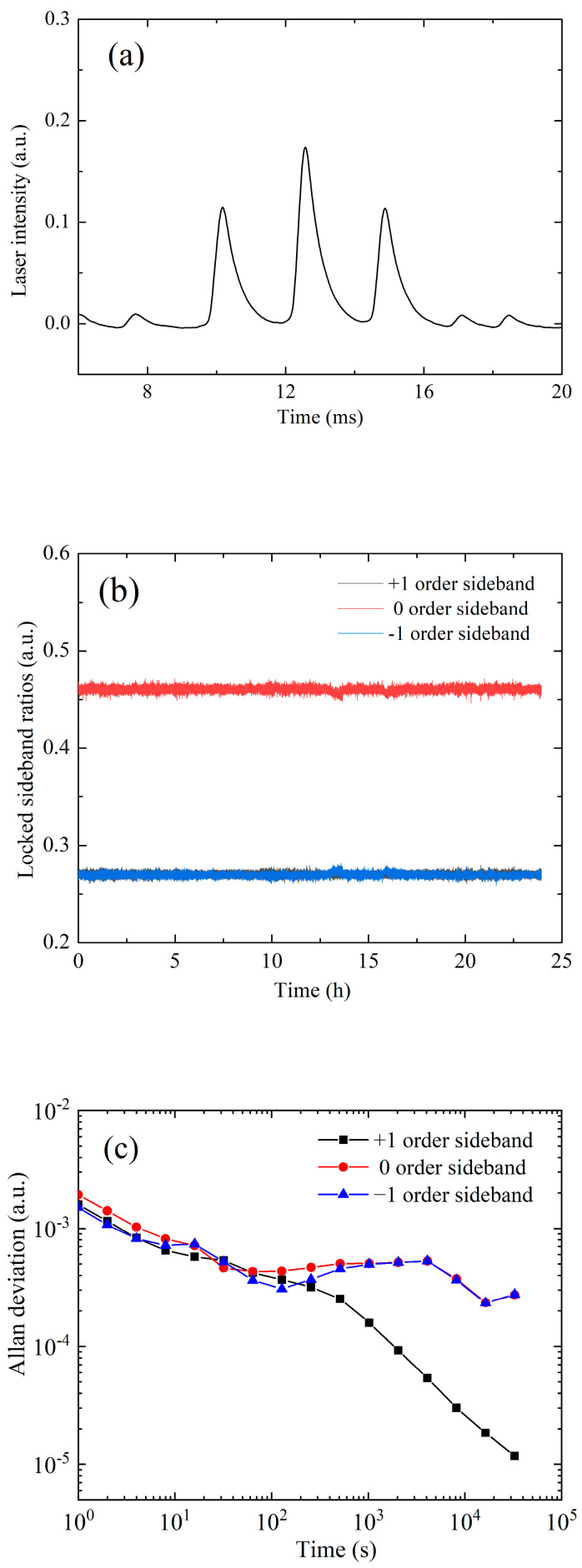
Experimental data for Raman laser sidebands. (**a**)The measured intensity of the sidebands during the Raman interference process by the FP cavity. (**b**) The measured −1, 0, 1 order sideband ratios after locking by two independent feedback loops. (**c**) The Allan deviation of the normalized locked sideband ratios.

Due to the fluctuation of the microwave power, and the nonlinear effect of the TA, the sideband ratio of Raman lasers will vary with time, and the sideband ratios $\beta_{+}=I_{+1}/I$ and $\beta_{-}=I_{-1}/I$ of the ± 1 order sidebands are not the same. We found a way to lock the values of these two sideband ratios. We measured the intensity of the sidebands during the Raman interference process by the FP cavity as shown in Figure 6a, and calculated $\beta_{-}$ and $\beta_{+}$ according to the measured intensities. We locked the $\beta_{+}$ to the target value by feedback of the microwave power of FEOM2, and locked the $\beta_{-}$ by feedback of the TA’s temperature; the locked sideband ratios are shown in Figure 6b, The Allan deviation of the sideband ratios is 2.5 × 10^−4^, the long-term stability of the gravity measurement is calculated to be less than 0.29 μGal.

### 4.3. Suppressing the Crosstalk of Lasers

The EOM scheme will produce positive and negative multi-sidebands, in addition to the Raman laser pair we need, other sideband combinations can also result in effective Raman transitions, thereby causing the phase and amplitude variation of the interference fringe, which is called crosstalk (the additional laser lines effect [28]).

This crosstalk depends on several experiment parameters, such as the velocity of the atom cloud *υ*_0_ at the time of the first Raman pulse, the free evolving time *T*, and the position *z* of the Raman mirror. If *υ*_0_ and *T* satisfy the following relationship, the variation of the phase and amplitude of the fringes is minimal.

(3) $$\upsilon_{0}=\left(m-\frac{n}{2}\right)\sqrt{\frac{1}{n}\frac{2\pi g}{\Delta k}},\;T=\sqrt{n\frac{2\pi }{\Delta kg}},$$

where, *m* and *n* are positive integers, Δk is the differential effective wave vector of the different Raman laser pairs, *g* is the gravitational acceleration. Table 1 lists the value of *υ*_0_ and *T* for different *n* and *m*. For our design, we consider both the size of vacuum chamber and the measurement precision, and choose *m* = 1, *n* = 1. The total time from releasing the atom cloud to the fluorescence detection is 214 ms, and the corresponding falling distance is 225 mm.

**Figure 7:**
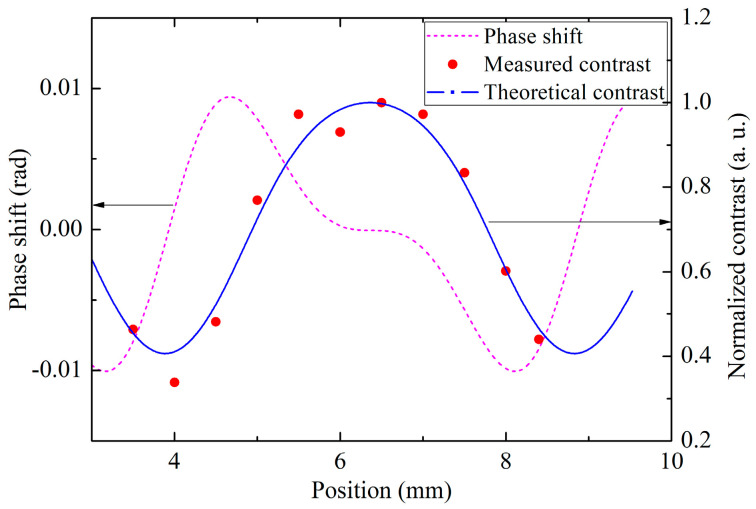
The contrast and phase shift of the interference fringe varying with the position of the Raman mirror *z*. The dashed line is the calculated contrast, the red dot-line is the measured contrast, and the pink solid line is calculated phase shift.

The position *z* of the Raman mirror also leads to a variation of the fringe’s phase and amplitude. Therefore, we designed a structure to adjust the position of the mirror in the vertical direction. Figure 7 shows the dependence of fringe contrast and phase shift on position of the Raman mirror. When the contrast is maximal, the phase shift is predicted to be zero. In the experiment, we adjusted the mirror to the position where the contrast is maximal with an accuracy of 1 mm, the corresponding phase shift caused by crosstalk is calculated to be less than 0.1 mrad, and the corresponding gravity measurement error is less than 0.12 μGal.

### 4.4. Vibration Compensation

**Figure 8:**
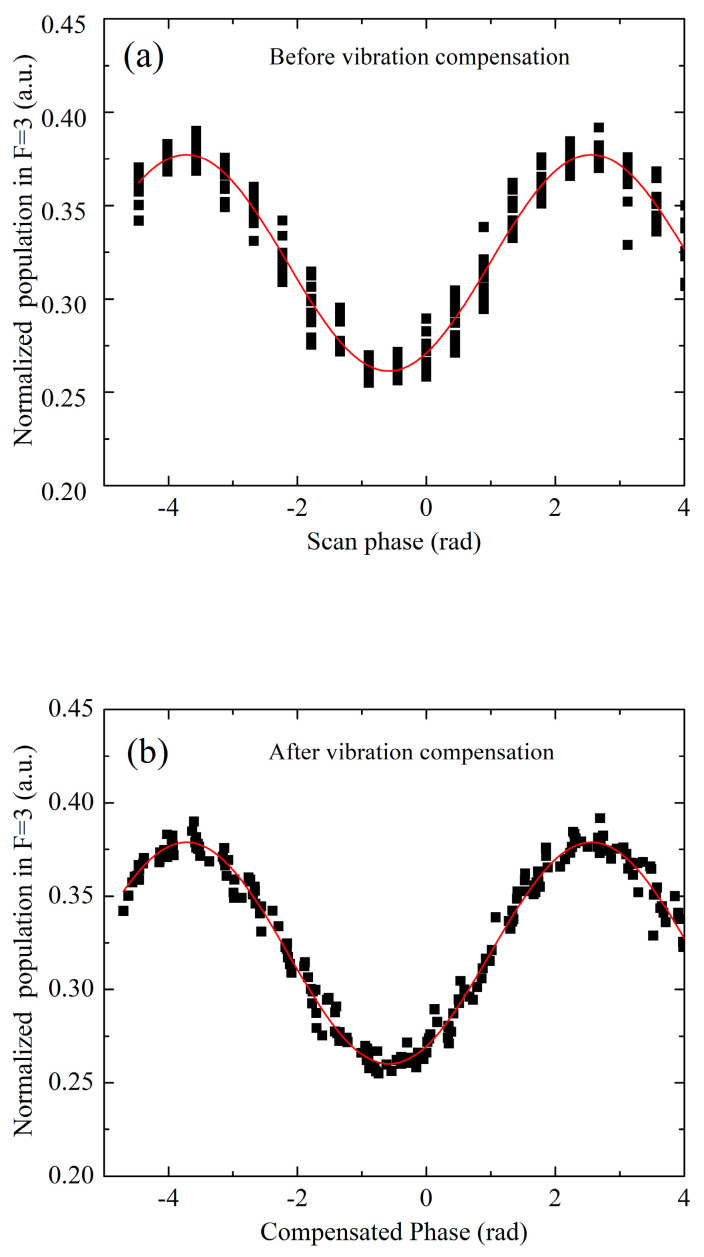
Interference fringe before (**a**) and after (**b**) the vibration compensation. Black square represents measurement point, each group of interference fringe consists of 20 points. Red solid line represents Sine fitting of the 10 groups of fringes.

To suppress the vibration-induced phase noise [29], we measured the vibration signals in the time interval of the interference process and compensated for them in the fringe fitting process. During the interference process, a seismometer mounted at the sensor head measures the velocity signal, and we calculated the phase shift of fringe according to the measured velocity and the phase sensitivity function. Then, we compensated for the phase shift and fit the interference fringe with a group of experiment data, which usually contains data from 20 experimental cycles. By using this method, the fitting residual phase noise of 10 groups of fringes is improved from 17 mrad to 10 mrad, as shown in Figure 8.

### 4.5. Gravity Measurements

**Figure 9:**
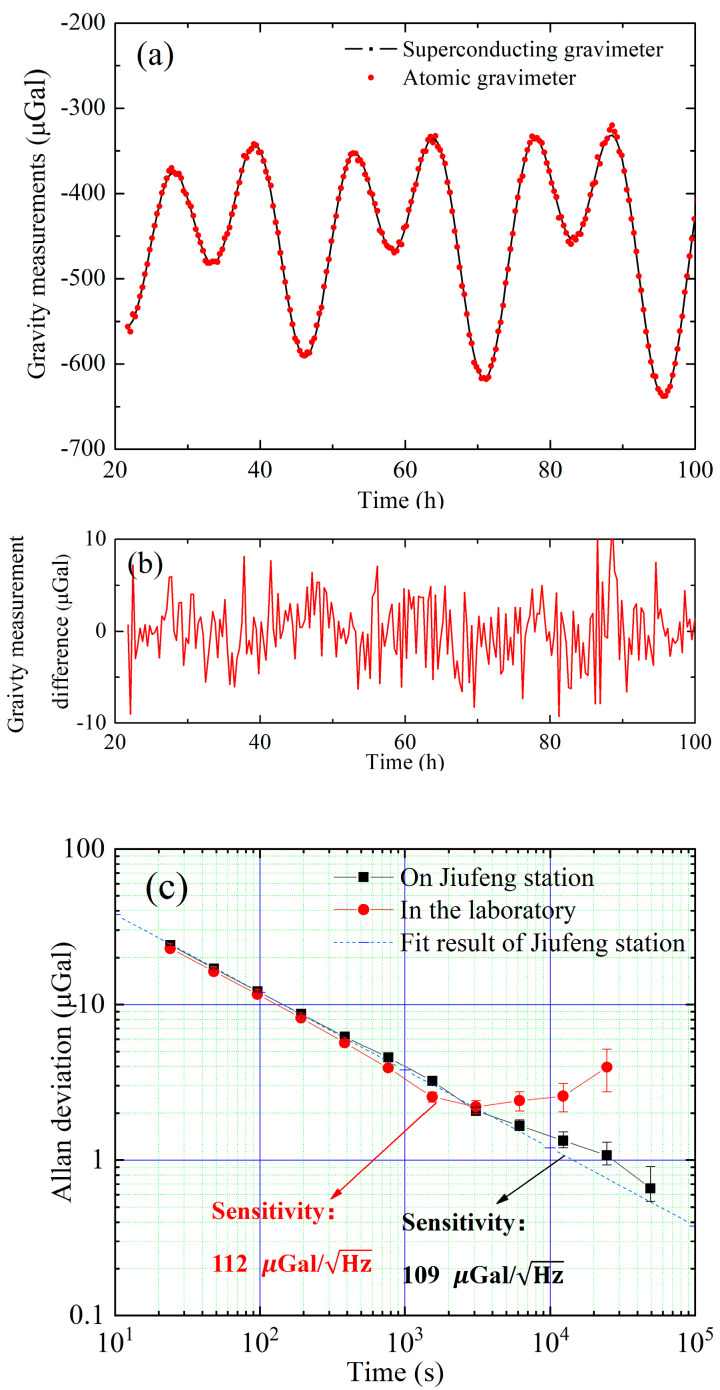
The gravity measurements and evaluation. (**a**) Gravity measurement and (**b**) Difference of measured gravity value between AG and the superconducting gravimeter. (**c**) Allan deviation of the gravity measurements.

We carried out gravity measurement in the laboratory to test the performance of the AG. In order to reduce high-frequency noise in the laboratory, we placed the AG on a vibration-isolated platform; at that time, we did not implement the double locking of the sideband ratio and automatic tilt adjustment features. The Allan deviation of the gravity measurement after subtracting the theoretical, calculated solid tide, is shown in Figure 9c. The short-term stability is 112 μGal/$\sqrt{\mathrm{Hz}}$. For time longer than 2000 s, the measurement value drifts; this is mainly induced by the inclination angle drift of the isolation platform.

Then, we transported the AG to the Jiufeng Gravity Observation Station in Wuhan, and refined and implemented the features of the double locking of the sideband ratio and automatic tilt adjustment features. We carried out long-term gravity observation, and compared the measured gravity value of AG with a superconducting gravimeter; the experimental results are shown in Figure 9. The superconducting gravimeter is a high-resolution, low-drift relative gravimeter with excellent long-term stability. The resolution of a station-based superconducting gravimeter can reach 1 nGal, with drift as low as a few μGal/year. The short-term stability of the difference gravity measurement is 109 μGal/$\sqrt{\mathrm{Hz}}$, and the long-term stability over 50,000 s is 0.65 μGal.

## 5. Conclusions

We developed a high-precision and miniaturized ^85^Rb AG with several specifical designs. A PZT-driven mirror mount is used to adjust and lock the direction of the Raman laser, and an electrical controlled bracket is used to automatically adjust the attitude of the sensor head. The calculated values are used to set and optimize the position of Raman mirrors and interference of the time sequence, thus suppressing the crosstalk and ac Stark shift of the multi-sideband of lasers. The long-term stability of the gravity measurement is better than 1 μGal. These results provide a reference for the implementation of high-precision portable AGs.

The short-term stability of the gravimeter is mainly limited by the residual vibration noise, which is mostly caused by the unknown transfer function between the seismometer and the Raman laser mirror. The residual vibration noise can be further suppressed by carefully calibrating the transfer function. The drift of the gravity measurement might be caused by the drift of the frequency of the Raman laser; this is because we used a saturated absorption spectroscopy with a Doppler background. When the power of the incident laser of the saturated absorption module changes, the demodulation signal might drift, and a Doppler free saturated absorption spectroscopy can be used to further suppress the frequency drift. Finally, to further improve the measurement accuracy, the system errors should be carefully calibrated.

## Figures and Tables

**Table 1:** Best velocity value of the atomic cloud *υ*_0_ at the time of the first Raman pulse and the interference free evolving time *T* for different value of *n* and *m*.

*n*	*m*	*T* (ms)	*υ*_0_ (cm/s)
1	1	71	34.8
1	2	71	104.3
2	2	100.4	49.2
2	3	100.4	98.4
3	2	122.9	20.1

## Data Availability

Not applicable.
